# Meta-Analysis of EMT Datasets Reveals Different Types of EMT

**DOI:** 10.1371/journal.pone.0156839

**Published:** 2016-06-03

**Authors:** Lining Liang, Hao Sun, Wei Zhang, Mengdan Zhang, Xiao Yang, Rui Kuang, Hui Zheng

**Affiliations:** 1 CAS Key Laboratory of Regenerative Biology, Guangdong Provincial Key Laboratory of Stem Cell and Regenerative Medicine, Guangzhou Institutes of Biomedicine and Health, Chinese Academy of Sciences, Guangzhou, Guangdong, China; 2 Department of Computer Science and Engineering, University of Minnesota Twin Cities, Minneapolis, Minnesota, United States of America; West China Second Hospital, Sichuan University, CHINA

## Abstract

As a critical process during embryonic development, cancer progression and cell fate conversions, epithelial-mesenchymal transition (EMT) has been extensively studied over the last several decades. To further understand the nature of EMT, we performed meta-analysis of multiple microarray datasets to identify the related generic signature. In this study, 24 human and 17 mouse microarray datasets were integrated to identify conserved gene expression changes in different types of EMT. Our integrative analysis revealed that there is low agreement among the list of the identified signature genes and three other lists in previous studies. Since removing the datasets with weakly-induced EMT from the analysis did not significantly improve the overlapping in the signature-gene lists, we hypothesized the existence of different types of EMT. This hypothesis was further supported by the grouping of 74 human EMT-induction samples into five distinct clusters, and the identification of distinct pathways in these different clusters of EMT samples. The five clusters of EMT-induction samples also improves the understanding of the characteristics of different EMT types. Therefore, we concluded the existence of different types of EMT was the possible reason for its complex role in multiple biological processes.

## Introduction

Investigations on epithelial-mesenchymal transition (EMT) and its reversed process, mesenchymal-epithelial transition (MET), can be dated back to 19^th^ century and significantly accumulated over the last several decades [1–3]. It is widely accepted that EMT plays important roles for cancer metastasis since the loose cell-cell interaction and high migratory ability resulted from EMT help the primary tumor cells detach from other cells or basement membrane and invade into blood vessel. Therefore, multiple chemical drugs against EMT have been developed for cancer therapy [2, 3]. However, two recent reports suggested that EMT is required for chemoresistance rather than metastasis in mice model with spontaneous multifocal breast adenocarcinomas and pancreatic cancer respectively [4, 5]. Such controversy not only further indicates the complexity of EMT but also supports the existence of different types of EMT. Charactering the gene expression changes during EMT should provide additional information to understand its complexity and diversity.

On the other hand, a sequential process including an early EMT and a late MET is also interesting. During cancer progression, MET has been reported after cancer metastasis or when cells re-gain epithelial characteristics at secondary tumor sites [6]. Multiple rounds of sequential EMT-MET also play important roles during embryonic development. The first round of sequential EMT-MET starts at gastrulation [7], and the proper development of heart requires multiple rounds of sequential EMT-MET [2, 8]. In addition, MET was also reported as an early and required step during the generation of induced pluripotent stem cells (iPSCs) from mouse embryonic fibroblasts (MEFs) [9, 10]. However, introducing the four factors in a particular sequence promoted iPSCs generation by inducing a short EMT before the MET [11]. The changes on cell morphology, cell mobility and gene expression also suggested a sequential EMT-MET during the conversion from MEFs to functional neurons [12]. These beneficial roles of such sequential EMT-MET are possibly linked to the concomitant between the changes on gene expression or epigenetic modification and the intermediate mesenchymal state. Thus, charactering the gene expression changes during EMT should also provide additional information to understand sequential EMT-MET during these cell fate conversions.

In previous studies, multiple investigations were performed to identify the conserved gene expression changes across different EMT. In 2012, a 130-gene list (referred as PO-list in following text) was identified from 18 published Gene Expression Omnibus (GEO) datasets to characterize EMT [13]. Thierty JP’s laboratory developed an EMT scoring system, which contains 315 gens and 218 genes (referred as EM-list in following text) as generic signature for tumor and cell line EMT [14]. In addition, 377 experimentally verified genes (referred as SC-list in following text) were manually curated from literature to develop a new database for EMT by Hong Qu’s laboratory recently [15]. These reports definitely facilitated our understanding on EMT except three remaining problems. Firstly, the overlapping of any two of these three gene lists was lower than expected, and seldom there were genes been identified in all of these reports. The low overlapping rates among different lists might be explained by the different levels of EMT induced in different samples or the fact that EMT could be classified into different types. In addition, the SC-list contains a large number of transcriptional factors, possibly because the genes were found from literature where EMT might be easily induced through the highly connected network of transcriptional factors. Secondly, epigenetic regulations including DNA methylation, histone methylation, acetylation and some other mechanisms during EMT have also been studied and reviewed extensively [16]. For example, HDAC inhibitors were useful in treating certain hematological tumors possibly by impairing EMT [17]. Histone demethylase LSD1 is also involved in EMT and cancer progression [18, 19]. Ten-eleven translocation methylcytosine dioxygenase 1 also regulates EMT by modulating DNA methylation [20, 21]. However, all the three meta-analyses identified few genes related to epigenetic modulation, possibly because the changes in protein activities can not be revealed in microarray analysis or the expression changes of these genes are not significant or conserved enough during different EMT. Thirdly, although study in human system is closer to clinical research on cancer and other fields than in mouse system, combining the study in both human and mouse system will be helpful.

In this study, 74 paired samples from 24 human microarray datasets and 31 paired samples from 17 mouse microarray datasets were analyzed together to identify generic signatures for EMT, and compared with the previous three reported lists, PO-list, EM-list, and SC-list. We clustered the paired samples and analyzed the clusters to demonstrate the possible existence of different types of EMT. In addition, we further validated the hypothesis by removing samples with weakly-induced EMT and functional analysis by Gene Ontology and pathways.

## Materials and Methods

### Expression profile data integration

All the datasets were downloaded from GEO. Searching “epithelial-mesenchymal transition” or “EMT” in GEO resulted in 144 human and 59 mouse microarray datasets. We first removed the datasets that focus on primary cancer sample/tissues because of the complexity in cancer initiation and the difficulty in identifying proper controls to determine the intensity of induced-EMT. Secondly, only the datasets for studying the EMT induced by transforming growth factor-β (TGFβ) or Snai1/Snai2/Twist over-expression were selected for further analysis, since it is widely accepted that these genes induce typical EMT [3, 22]. The other datasets for studying EMT induced by serum starving, p53 knockdown, cell density, overexpression of Notch, Six1, Runx2 or other genes that are not widely accepted as EMT inducer were not included in current analysis. The detailed information of the selected dataset (24 human and 17 mouse datasets) such as assigned GSE number, cell types, treatment details, and reference (if available) were listed in S1 Table. The normalized intensity of all the probes (from series matrix files) and the matched GPL platform files with the probe annotations of each microarray dataset were downloaded from GEO. After pooling together, the normalized intensity of each probe is reported in each sample in the datasets,

The biological replicates of the same treatment were averaged and further normalized with the control samples. If there are multiple treatments to induce EMT in one dataset, the same control sample was used. In the two-channel array datasets, dual fluorescence dyes are labeled in both treatment and control samples for the ratio, the normalizing step was omitted for these datasets. Finally, the probes measuring the expression of the same gene were averaged in all the samples.

To match gene symbols and their aliases across the datasets, we downloaded the official symbols and all alternative symbols of mouse genes classified as “protein coding genes” from Mouse Genome Informatics (MGI, http://informatics.jax.org/)). The same list for human genes was downloaded from HUGO Gene Nomenclature Committee (HGNC, http://www.genenames.org/). The gene names in each dataset were replaced with their official symbols in the downloaded annotations. The gene names that were not included in the two lists were manually searched in NCBI and Ensemble datasets to identify their official symbols. The datasets were merged based on the official symbols. The results were then transformed into log_2_ values. Finally, the data tables (S2 and S3 Tables) with 74 and 31 EMT-induction samples/pairs for human and mouse respectively were generated.

### Clustering gene expression profiles

Experiments with similar log-ratio expression profiles were grouped together by applying the Hierarchical Clustering method for both human and mouse data. In the analysis, only the genes with expressions data available across all the samples were applied to build up the hierarchical tree. The correlation distance (1 minus the correlation) was utilized to find the dissimilarity between every pair of samples in the integrated datasets. The samples were merged in close proximity using the farthest neighbor clustering method in linkage criteria, i.e. complete linkage. In each iteration, the current clusters were further grouped into larger clusters until the complete hierarchical tree was formed. In the hierarchical tree, the samples with more similar expression profiles are connected by deeper branches. The hierarchical clustering results for human and mouse data were used for further analyses. The pairwise correlation coefficients matrices are shown under the hierarchical trees.

To further validate the hierarchical clustering, a consensus clustering method that has been widely applied to gene expression data analysis for robust clustering was also used to cluster 74 human and 31 mouse EMT-induction pairs [23].

### EMT generic signature identification

Significant gene expression changes (p-value<0.05) were identified by analyzing log_2_ values of expression changes in selected EMT-induction samples with two-tailed student t-test. The t-test was applied to all the EMT-induction samples to identity the overall generic signature and the samples in each cluster to identify cluster-specific signature. The significant gene expression changes were identified with human and mouse EMT-induction samples separately. To identify EMT generic signature conserved in both human and mouse, the mouse gene symbols and human gene symbols were converged to match their homologous genes (if available) with the annotation from MGI. In the comparison, 579 genes up-regulated/down-regulated in 31 mouse and 74 human EMT-induction samples were summarized in S4 Table.

Significant gene expression changes in human clusters were identified with slightly modification in order to identify similar number of expression changes in each cluster. For example, in addition to a smaller p-value less than 0.05, the average of the log_2_ values in Cluster I should be over 0.585 or below -0.585 (over 150% or below 67%). The p-value criteria for the other four clusters was also 0.05, while the expression criteria were ±0.585,±1.322 (>250% or <40%), ±0.585, and ±0.322(>125% or < 80%) for Cluster II, III, IV, and V, respectively.

### Evaluation of EMT induction

PO-list contained 130 genes and was obtained from the EMT generic signature identified previously [13]. EM-list contained 416 genes and was combined from the generic signature for cancer and cell lines [14]. The 377-gene SC-list was also reported previously by manual curation from literature [15]. The three lists were further combined into an 805-gene list. Since microRNAs and several other genes were not available in the integrated data, a smaller 773-gene list was used for further analysis combined from 128, 407 and 357 genes from PO-list, EM-list and SC-list after removing the micro-RNAs, respectively (S5 Table).

To derive the levels of induced EMT in each sample, four scoring systems were used to evaluate the induced EMT. The expression changes of 128 genes (66 mesenchymal and 62 epithelial genes) in PO-list and 407 genes (184 mesenchymal gene and 223 epithelial genes) in EM-list were used to score each EMT-induction sample (S6 Table). In addition, the 107 genes identified in at least two of those three lists, PO-list, EM-list and SC-list, were used as another scoring system (S6 Table). Finally, since *Cdh1* and *Cdh2* are widely accepted EMT markers and have been identify in almost all lists [1, 11], the up-regulation of *Cdh2* and down-regulation of *Cdh1* during EMT were used as the forth scoring system to evaluate the induced EMT in different samples. The four scoring systems were used independently and weighted equally during the evaluation.

Four scoring systems based on different gene lists were used to score each EMT-induction sample separately. The four gene lists were, 128 genes in PO-list, 407 genes in EM-list, 107 genes identified in at least two of those three lists, and the gene *Cdh1/2*. The SC-list is not included because the gene list was curated from literature instead of being derived from experimental data. The genes in all the four scoring system were classified as mesenchymal genes and epithelial genes depending on previous reports [13, 14]. For each scoring system, the average log_2_ values of mesenchymal gene expression changes minus the average log_2_ values of epithelial gene expression changes to obtain the score for evaluation. The scores in each evaluation system were further normalized by the average score of all EMT-induction samples, and the final score for each EMT-induction samples was generated by averaging the four normalized scores. Genes in these four scoring system were used to evaluate mouse EMT-induction samples only if they have homologous between human and mouse basing on homologous information from MGI.

The EMT-induction samples with a final score lower than 0.10 were classified as samples with weakly-induced EMT, while the other classified as samples with strong or medium EMT by a threshold of 0.45 in human and at 0.70 in mouse.

When evaluating the EMT during neuron trans-differentiation, the scores of all 31 mouse EMT-induction samples were further normalized by the mean into comparable scores among mouse EMT-induction samples.

Overlapping score between EMT during neuron trans-differentiation and those in one of the five clusters was calculated by subtracting the absolute log_2_ values of gene with opposite expression changes from the summary of genes with consistent expression changes.

### Gene ontology (GO) analysis

GO and Kyoto Encyclopedia of Genes and Genomes annotation analysis were performed with DAVID (https://david.ncifcrf.gov/) [24, 25].

### Statistical analysis

The statistical analysis was performed with GraphPad Prism 5.0. The build-in packages for two-tailed student t-test, one-way ANOVA with Dunnett’s test as a post-hoc, and Fisher’s Exact test were used.

## Results

### Data collection and GO analysis of generic signatures

To identify the generic signature of EMT, 24 human and 17 mouse microarray datasets were downloaded from GEO and processed as described in Materials and Methods (S1 Table). After integration, the integrated datasets contain 74 and 31 EMT-induction samples from human and mouse datasets respectively (S2 and S3 Tables). The human EMT-induction samples included 18060 genes annotated as protein coding genes by HGNC. The 18060 genes were covered among 85% of the 74 EMT-induction samples on average and more than 88% of the 18060 genes were covered in more than 44 samples (>60%) (S2 Table). The mouse EMT-induction samples included the 20963 genes annotated as protein coding genes by MGI. The 20963 genes were covered among 85% of the 31 samples on average and more than 80% of the 20963 genes were covered in more than 18 samples (>60%) (S3 Table).

**Fig 1:**
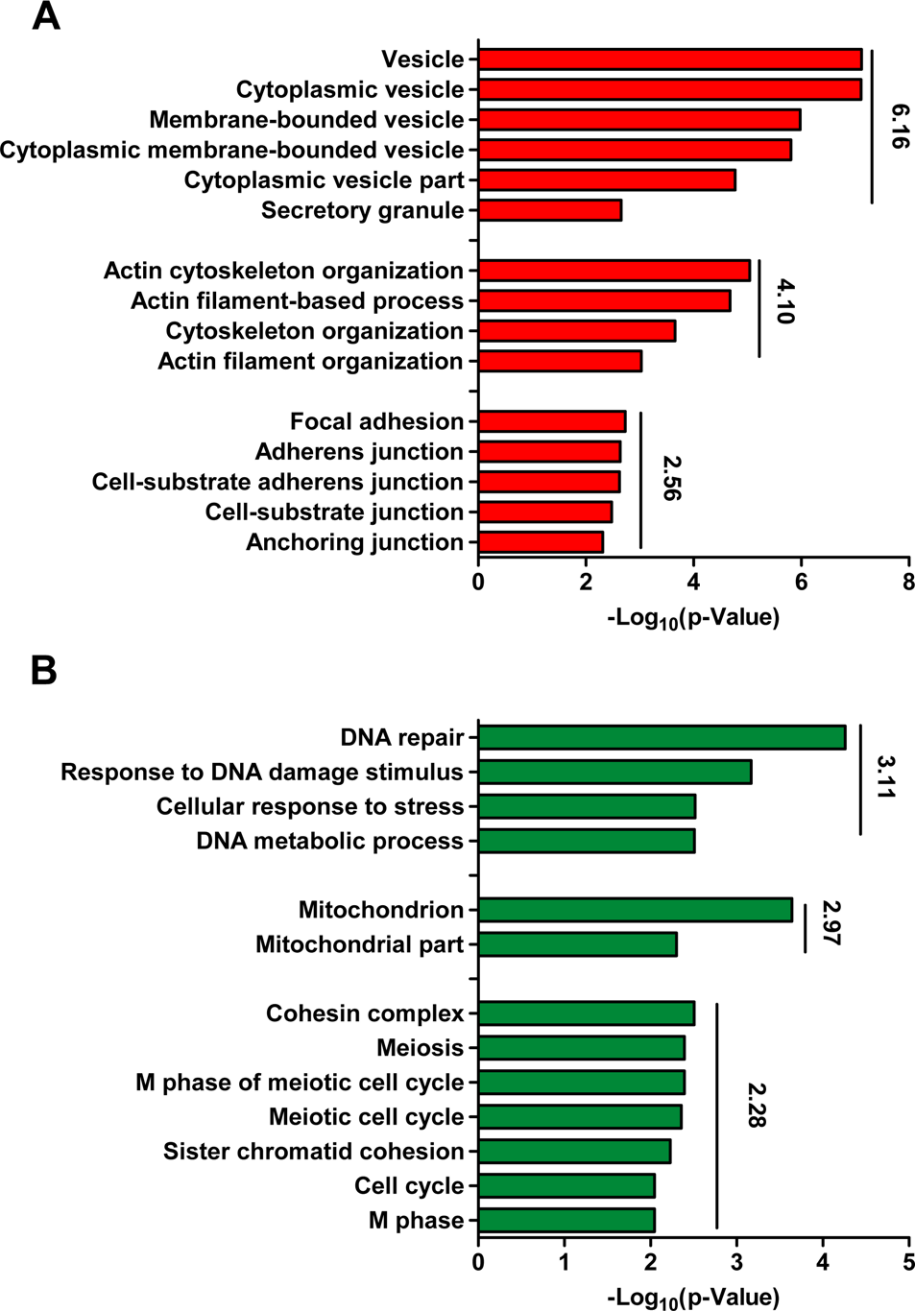
GO analysis with identified gene signatures. 579 genes were identified after analyzing 74 human and 31 mouse EMT-induction samples as described in Materials and methods. 378 up-regulated and 201 down-regulated genes were subjected to GO analysis, and enriched GO terms were listed in (A) and (B). The average–log_10_ (p-Value) is shown next to the bar plots.

Based on the homology annotation in MGI, 15870 homologous genes between human and mouse were subjected to further analysis. 378 up-regulated genes and 201 down-regulated gene were identified as generic signatures for EMT, including wildly accepted EMT markers like *Cdh2*, *Fn1*, *Tgfb1/2*, and *Zeb2* (S4 Table). GO analysis suggested that the up-regulated genes enrich cytoplasmic vesicle, cytoskeleton organization and cell-junction, while the down-regulated genes enrich DNA repair, cell cycle and mitochondrion envelop (Fig 1A and 1B).

### Comparing the detected generic signature with reported EMT-related gene lists

The list of the detected generic signature was then compared with three previous reported lists [13–15]. For convenience, the three previous gene lists were referred as PO-List [13], EM-list [14] and SC-list [15] labeled by the journals publishing the articles. An 805-gene list was generated by combining the genes in EM-list, PO-list and SC-list together. This list was then shortened to a 773-gene list (773-list) because microRNAs and several other genes were not available in the integrated data (S5 Table). Low overlapping rates were observed between the identified 579-gene list and the three previous lists with 12, 39 and 28 genes in common in PO-list, EM-list and SC-list respectively (Table 1). Furthermore, similar low overlapping among the three previous lists was also observed (Table 1). Only 12 genes, *Cd24*, *Cdh1*, *Cdh2*, *Epcam*, *Fn1*, *Lox*, *Mmp2*, *Ocln*, *Prss8*, *Vcan*, *Vim*, and *Zeb1*, were in common in all the three lists and only 95 genes were in common in at least two of the three lists (Table 1).

**Table 1:** The overlapping among EMT generic signature lists.

	Current(579)	PO-list(128)	EM-list(407)	SC-list(357)
Current (579)		12	39	28
PO-list (128)			61	46
EM-list (407)				24
SC-list (357)				

579 genes were identified after analyzing 74 human and 31 mouse EMT-induction samples as described in Materials and methods. These 579 genes were compared to the genes in three previous lists, PO-list, EM-list, and SC-list. The numbers of genes identified in any two of these lists were listed.

Between all these four lists of EMT generic signatures, the highest overlap is observed between PO-list and EM-list. The 61 overlapping genes account for about 47% and 15% in the two lists (Table 1). In addition, although PO-list had higher overlapping with the other lists, the overlapping with the current 579-gene list was not significantly different from EM-list and SC-list. Thus, the generic signatures generated from different investigations could not be successfully reproduced by each other, which further suggested generic network employed by EMT could be more complex than expected.

### Levels of EMT is not responsible for the low reproducibility

There were three possible explanations for the low overlapping among the lists. The first possible explanation was that EMT was induced to different levels in the samples. The different levels of induced EMT could obscure the gene expression changes during EMT such that the changes are not detectable in certain samples. The discrepancy subsequently impaired the identification of conserved gene expression changes. Based on the reasoning, removing the samples with weakly-induced EMT should increase the agreement among the lists.

74 human and 31 mouse EMT-induction samples were classified into three categories, strong, medium and weak EMT induced, based on their scores (S6 Table). Another three lists of EMT generic signature were then generated from the Strong-list, Medium-list and Weak-list by the classification with the scoring systems (S7 Table).

**Fig 2:**
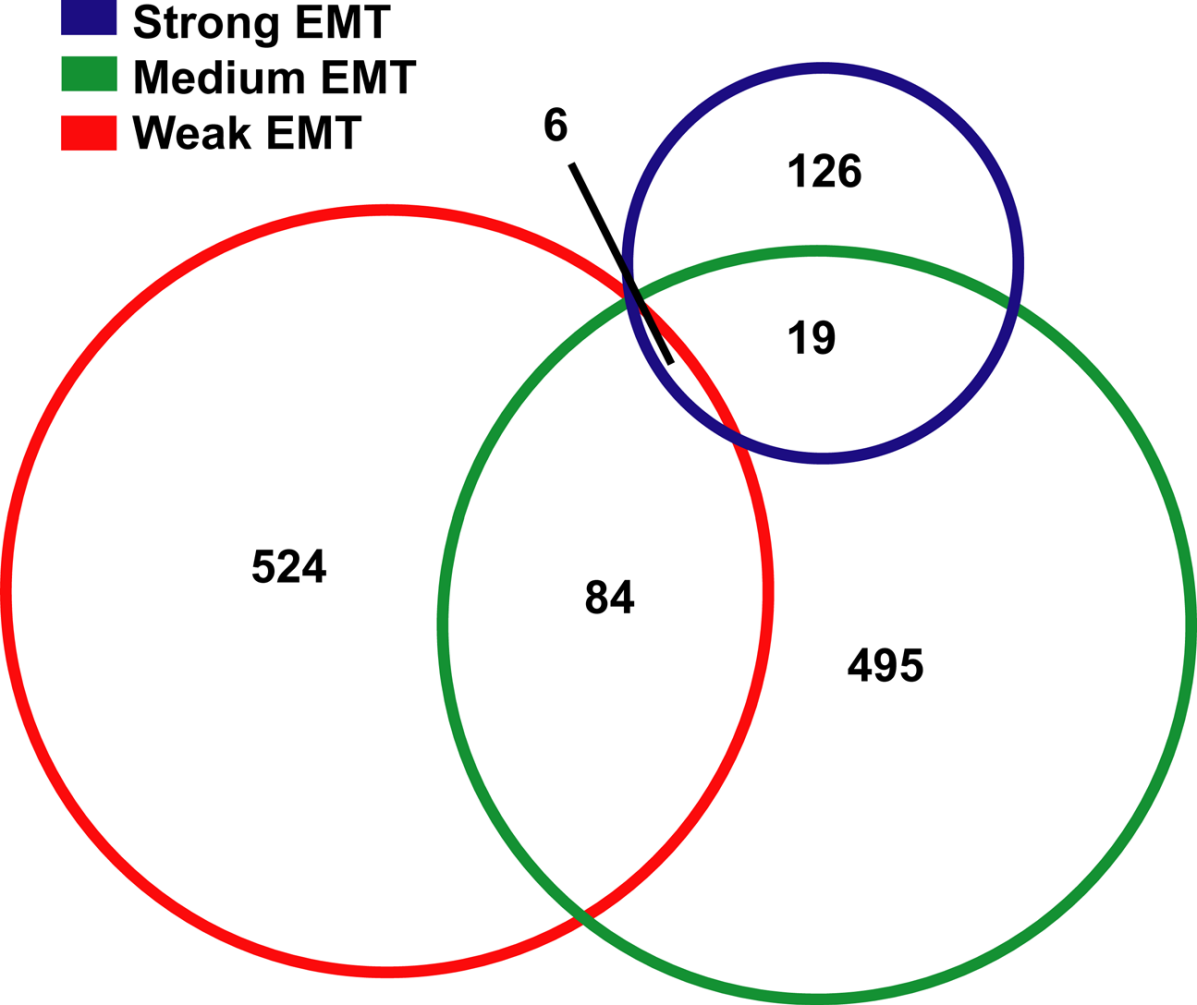
Low overlapping among samples with different EMT induction. 74 human and 31 mouse EMT-induction samples were evaluated with four scoring systems and classified into three groups, samples with strong EMT, medium EMT or weak EMT. Significant gene expression changes were identified in these three groups. The overlapping of genes in these three lists, Strong-list, Medium-list and Weak-list, were indicated.

The overlapping among the three new lists was also low. Only six genes were identified in all the three lists and 86 genes were identified in two of the three lists. The remaining 955 genes (over 91%) were only identified in one list (Fig 2). The three gene lists were also compared to PO-list, EM-list and SC-list (Table 2). While the numbers of overlapping genes increased when the level of induced EMT increased, the difference was not significant. The overlapping between PO-list and Strong-list and Weak-list were 11 in 524 and 0 in 126, respectively, with a p-value at 0.1362. The similar comparison with EM-list and SC-list returns p-values at 0.3300 and 0.6337 (Table 2). Therefore, although these three lists were generated from samples with distinct levels of induced EMT, their overlapping with PO-list, EM-list and SC-list were not significantly different from each other (Table 2). In addition, after combining the Strong-list, Medium-list and Weak-list together and forming a 1047-gene list, the combined list was also quite different from PO-list, EM-list and SC-list (Table 2), which further suggested that the different levels of EMT should not completely account for the low overlapping.

**Table 2:** Removing weakly-induced samples does not improve overlapping.

	PO-list (128)	EM-list(407)	SC-list(357)
Strong-EMT (524)	11	25	26
Medium-EMT (495)	4	14	20
Weak-EMT (126)	0	3	4
Combined (1047)	13	38	45

74 human and 31 mouse EMT-induction samples were evaluated with four scoring system to determine the degrees of EMT induced. The resulted scores were used to classified the samples into three classes, Strong-EMT, Medium EMT, and Weak-EMT. EMT generic signatures were identified in these three classes, and the resulted lists of genes were compared with PO-list, EM-list, and SC-list. The numbers of genes identified in any two of these lists were listed below. Strong-EMT, Medium EMT, and Weak-EMT were combined to form a new 1047-gene list whose overlapping with three previous lists was in the fourth row.

### Cluster analysis of EMT samples

If removing the samples with weakly-induced EMT did not improve the overlapping significantly, the different levels of EMT-induction should not be the major reason for the low overlapping rates. Thus, we further investigate another possibility that there were multiple types of EMT. Although the different types of EMT shared some generic signatures like *Cdh1/2*, *Fn1*, and *Epcam*, which are highly linked to cell adhesion and cell mobility, each type might engage distinct mechanisms and pathways involved in the EMT.

**Fig 3:**
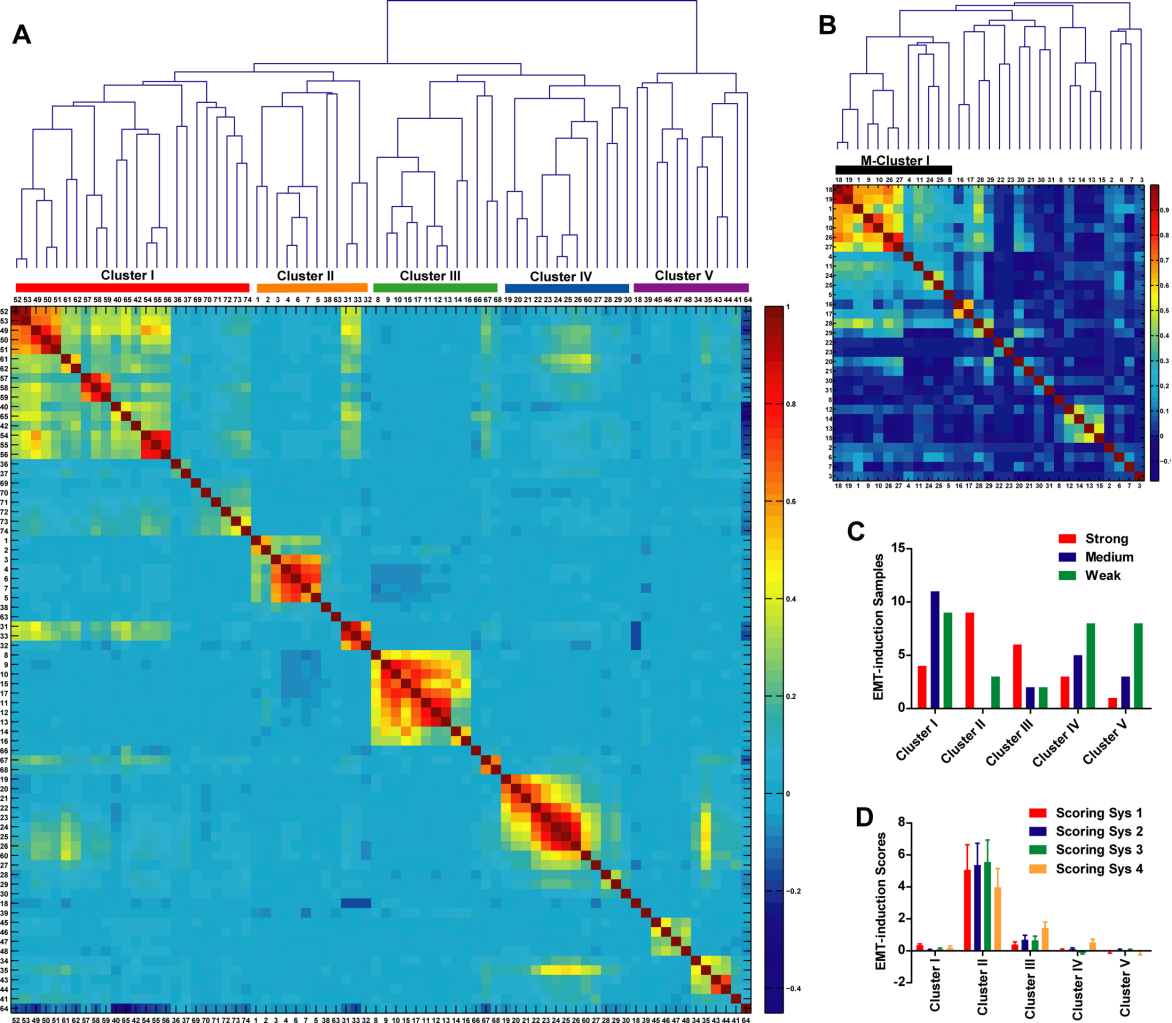
Clustering EMT-induction samples. (A-B) 74 human and 31 mouse EMT-induction samples were grouped into clusters by the expression profiles. The correlations among pairs of samples were shown in (A) for human and (B) for mouse. (C-D) 74 human EMT-induction samples were grouped into five clusters. The numbers of samples with strong EMT, medium EMT or weak EMT were listed in (C). The average scores of samples in these five clusters were listed in (D).

To test the hypothesis mentioned above, the 74 human and 31 mouse EMT-induction samples were subjected for clustering analysis. As indicated in Fig 3A and 3B, the EMT-induction samples were clearly clustered into several distinct groups. The 74 human EMT-induction samples were classified into five clusters. When the EMT-induction levels in these five clusters were measured, all the five clusters contain mixed samples with both strong and weak EMT induction (Fig 3C and S6 Table). Specifically, only cluster II was found to be dominated by strong EMT induction, while the other four clusters were not significantly different from each other (Fig 3D and S6 Table). Thus, the clustering of EMT-induction samples seemed to be independent of the levels of EMT induced overall. In addition, when considering the treatment and cells lines used for EMT induction in these samples as summarized in S1 and S6 Tables, no significant contribution of these factors to the current clustering was observed.

To further confirm the clustering results, consensus clustering [23] which is applied to the analysis of gene expression data for robust clustering was also used. As indicated in S8 Table, the five clusters identified with hierarchical clustering were also identified by consensus clustering. In particular, in the six clusters (cluster 1–6) identified by consensus clustering with k = 6, 22 out 24 Cluster I samples were clustered into cluster 5 (23 samples), and all 12 samples in Cluster II was clustered into cluster 1 (totally 12 samples). Samples in Cluster III was clustered exclusively into cluster 2. Cluster IV overlaps with the union of cluster 4, 5 and 6 and cluster V strongly overlaps cluster 3 and a few samples in cluster 4. We observed that samples in Cluster IV and V tend to be clustered together by both clustering method under different choice of k. Overall, we concluded that the five clusters identified with hierarchical clustering were consistent with consensus clustering. Types of cells, to some extent, may contribute to EMT induction. As summarized in S1 Table, 46.34% human mammary cells (19 in 41) clustered into cluster I, all human retinal pigment epithelium (RPE) cell line, APRE19, clustered into cluster III, while lung cells mainly clustered into cluster IV and V. All these suggested that cell types and/or the original state of cells may influence EMT induction. Since only 74 human samples were analyzed in our study, the correlation between cell types and EMT induction needs to be confirmed by more samples and further studies.

By using a modified strategy (Materials and Methods), the five clusters of human EMT-induction samples were used to establish five gene lists, which contained 2631 genes together with only about 13% genes been identified in more than one cluster (S9 Table). The low overlapping rates among the genes identified in these five clusters further confirm that the current clustering could reveal some true biological signals among these EMT-induction samples. In comparison between these generic signatures identified in these five clusters with PO-list, EM-list and SC-list, various extent of agreement are observed. The list generated from Cluster III had higher overlapping with PO-list than those from other clusters except Cluster II, while had higher overlapping with EM-list and SC-list than all four other clusters (Table 3).

**Table 3:** Five clusters of human EMT-induction samples overlap differently with other lists.

	PO-list (128)	EM-list (407)	SC-list (357)
Cluster I (623)	37	123	99
Cluster II (651)	72	132	77
Cluster III (603)	82	223	198
Cluster IV (711)	37	141	117
Cluster V (433)	19	48	43

74 human were clustered into five different clusters and used to identify five different lists of EMT generic signature. The overlapping of these five new lists with PO-list, EM-list, and SC-list were analyzed.

### GO term and KEGG pathway analysis of the generic signatures of five clusters

**Fig 4:**
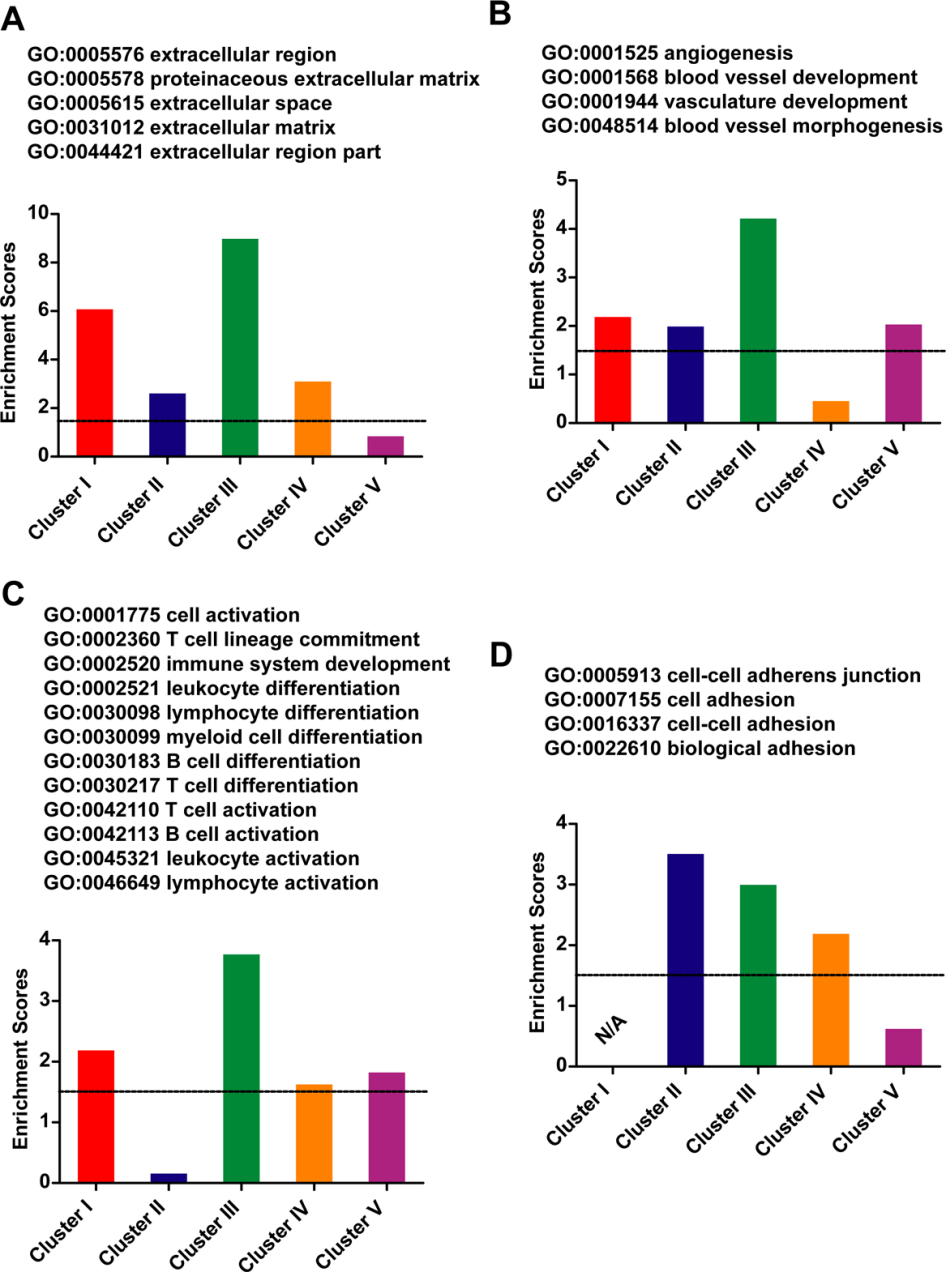
GO terms enriched in at least three clusters. Five clusters of human EMT-induction samples were used to identify five gene lists, which were subjected to GO analysis. The GO terms enriched in at least three clusters were listed. Averaged enrichment scores for related GO terms were represented. (A) extracellular matrix with five GO terms. (B) angiogenesis with four GO terms. (C) T/B cell activation with twelve GO terms. (D) cell adhesion with four GO terms.

The 2631 genes identified in each cluster were then subjected for GO and KEGG analysis. The GO annotation terms with significant enrichment in any one of the five clusters were then used for further analysis. Although there were only about 13% of the 2631 genes been identified in more than one cluster, there were several GO terms with significant enrichment in at least three clusters (Fig 4 and S10 Table). These GO terms were on extracellular matrix, angiogenesis, B/T cell activation, and cell adhesion, where the terms were consistent with the critical roles of cell-cell contact and cell mobility during EMT [3, 26, 27]. In addition, ECM-receptor interaction (KEGG: 04512) was also enriched in cluster I, II and IV.

**Fig 5:**
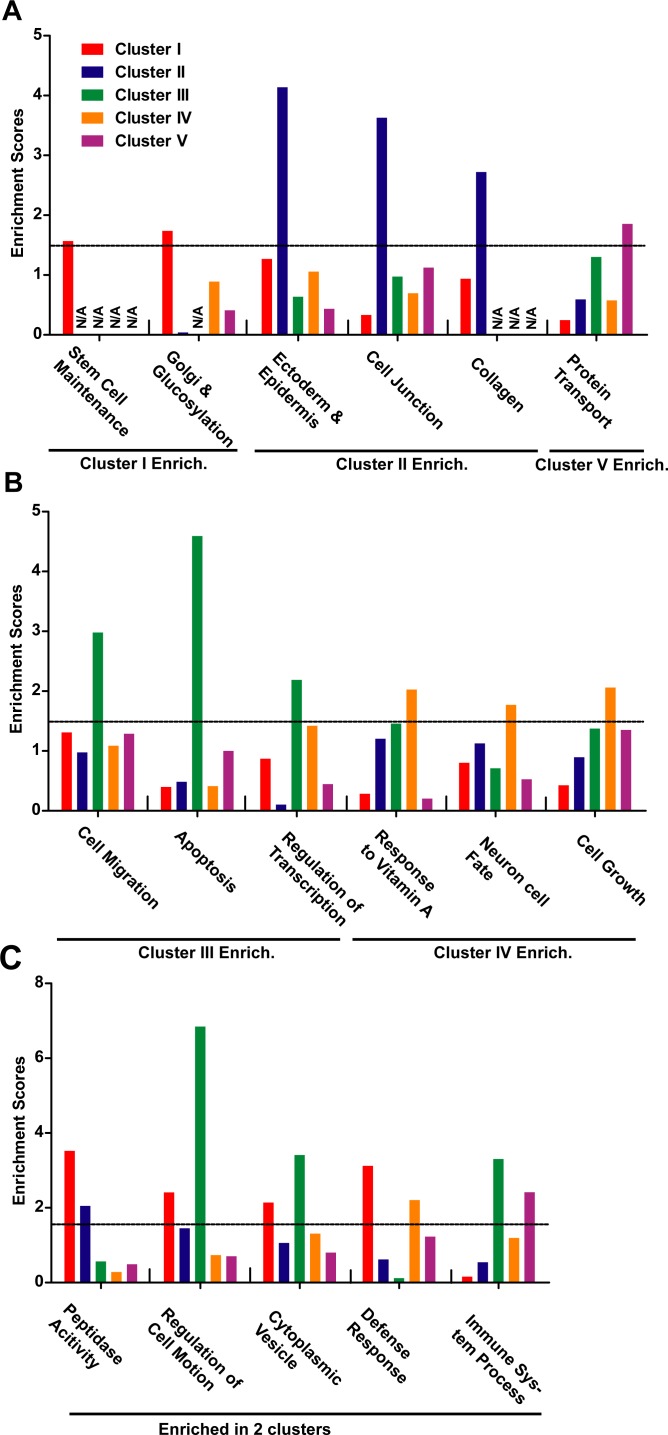
GO terms enriched in one or two clusters. Five clusters of human EMT-induction samples were used to identify five gene lists, which were than subjected for GO analysis. The GO terms enriched in Cluster I, II or V were listed in (A), while those enriched in Cluster III or IV were in (B). The GO terms enriched in two of the five clusters were listed in (C). The enrichment score for one group of GO terms were calculated by averaging the scores of included GO terms, which were provided by DAVID. Enrichment scores over 1.30 were considered as significantly enriched.

The genes identified in the five clusters of human EMT-induction samples also revealed significant differences in the enriched functions and pathways. The enrichment analysis by GO and KEGG suggested that genes identified in Cluster I were enriched on stem cell maintenance, glucosylation and glycosphingolipid biosynthesis (KEGG) (Fig 5A), possibly related to the increase in stemness during EMT and the roles that glycoproteins play during EMT [28]. The significant expression changes on genes like *Sox2*, *Eed* and *Sp1* were only identified in Cluster I. Since Sox2 highly expresses in stem cells such as pluripotent stem cells and neuron stem cells, we hypothesized this Cluster I EMT as stemness EMT, which will provide new information for understanding the roles that EMT play during cancer progression and generation of iPSCs [6, 11].

Genes identified in Cluster II were enriched on ectoderm and epidermis development, and cell junction (Fig 5A). *Cdh1*, *Fn1*, and *Tuba1a* were identified in this cluster, suggesting this EMT was highly related to re-organization of cell-cell contact. In addition, samples in Cluster II have the highest EMT scores than samples in other clusters (Fig 4D), further suggesting the EMT represented by Cluster II focused on the most basic changes during various EMT, in re-organization of cell-cell contact. Considering the loss of tight cell-cell contact and increase in cell migration, we considered this type of EMT as dissociation EMT.

Cluster III had higher overlapping with PO-list, EM-list, and SC-list than other clusters (Table 3), suggesting the EMT represented by Cluster III might be the most widely observed type of EMT. Genes identified in this cluster was enriched in cell migration, apoptosis, and regulation of transcription (Fig 5B). In addition, KEGG analysis suggested enrichment in several signaling pathway, TNF signaling pathway (04668), NOD-like receptor signaling pathway (04621), NF-κB signaling pathway (04064), and Chemokine signaling pathway (04062). Significant expression changes on *Traf1/2*, *Nfkb1/2*, *Cxcl1/5* and *Vcam1* were only identified in Cluster III. Thus the observations strongly suggest that the EMT represented by Cluster III mediated the transduction from outside stimulation to intracellular response.

Genes identified in Cluster IV were enriched in response to vitamin A, neuron fate specification, and regulation of cell growth (Fig 5B). Since vitamin A or its metabolic product retinoic acid can induce neuronal differentiation of pluripotent stem cells [29, 30], it is reasonable to consider this type of EMT as neuronal EMT. Similar EMT might be observed in processes that relate to neuron fate specification, such as trans-differentiation from fibroblast to neurons[12], which will be discussed in the following text. In addition, *Rara*, *Bmp2/4*, *Foxa1*, *Nkx6-2* and *Hes5* were identified in Cluster IV, further suggesting that this type of EMT might represent the ability of EMT to induce neuron fate specification.

Genes identified in Cluster V were enriched in protein transport and immune system process (Fig 5A). Actually, the samples in Cluster V were not well clustered compared to the other four clusters (Fig 3A). These samples were also not well clustered by consensus clustering (S8 Table). Although immune response is an important aspect of EMT, it is more appropriate to consider this type of EMT miscellaneous EMT.

Several GO terms including peptidase activity, regulation of cell motion, cytoplasmic vesicle, defense response and immune system process, were enriched in two of the clusters (Fig 5C). In summary, these five clusters of samples represent five different type of EMT: stemness EMT, dissociation EMT, signaling EMT, neuronal EMT, and miscellaneous EMT.

### Relevance of the identified GO and KEGG pathways with EMT

To verify the accuracy of the clustering results, the enriched GO terms and KEGG pathways in each cluster in EMT were further investigated. It was widely accepted that EMT confers epithelial cells stem-cell characteristics, and stem cells exhibited mesenchymal morphology and expressed markers associated with EMT [31, 32], and EMT was recognized as characteristic of CSCs [33, 34]. In addition to expression levels, the glycomic profile of human retinal pigment epithelial cells, malignant and premalignant epithelia underwent a profound reorganization upon EMT [35, 36]. Such changes may alter the interaction between cells and other cells or ECM. To alleviate intercellular adhesion during EMT, glycosphingolipid metabolism was also regulated, and in turn, glycosphingolipids may serve as mediator of EMT inducers, like Zeb1 [37, 38].

The GO and KEGG pathways identified in cluster II confirmed the integral role of EMT during development, and represent the processes directly related with morphological changes because they involves alterations in cell adhesion and cell mobility. They may reflect some most essential features of EMT, because the loss of tight cell junction was used to define EMT, and such features were only sufficient and significant by high levels of EMT. The changes in these pathways during weak and medium EMT may not be significant enough and thus undetectable.

Various signaling pathways can induce EMT. TGF-*β* pathway, Notch, Wnt/*β*-catenin, NF-*κ*B, RTKs, Hedgehog were reported to regulate and mediate EMT, and usually EMT was induced by the crosstalk of several pathways [39–42]. In cluster III, several other pathways were identified. The NOD-like receptor (NLRP) plays an important role in intracellular ligand recognition[43], and NLRP signaling pathway is the phylogenetically oldest sequence of pro-inflammatory gene expression and activation of the encoding proteins[44]. Chemokines play important role in EMT. For example, chemokine ligand 17 (CCL17), promotes the stemness, EMT process, the TGF-β1 and Wnt/β-catenin signaling in MHCC97L cells[45]; chemokine (C-X3-C motif) ligand 1 (CX3CL1), participates in the molecular events that regulate cell adhesion, migration and survival of human prostate cancer cells[46].

The expression analysis of cluster IV indicates the role of EMT in neuronal differentiation. During the development of cerebral cortex, E- and N-cadherin-mediated adherens junctions and gap junctions were observed in the pseudostratified neuroepithelium, which is composed of neural progenitors[47, 48]. Suppression of N-cadherin caused premature neuronal differentiation[49]. Neural crest cells, induced near the junction of the neural plate and embryonic ectoderm, undergo several EMT to migrate to desired places and differentiate into various types of neurons[50].

Upon EMT induction, cells remodeled the way they arrange themselves, and secreted proteins to dissociate their interactions with other cells and extracellular matrix. All these necessitate the activation of cytoskeleton and the endocytic and exocytic vesicle trafficking pathways to transport newly synthesized or existing proteins to the right locations[51].

### Clustering of EMT-induction samples facilitates the understanding of EMT

**Fig 6:**
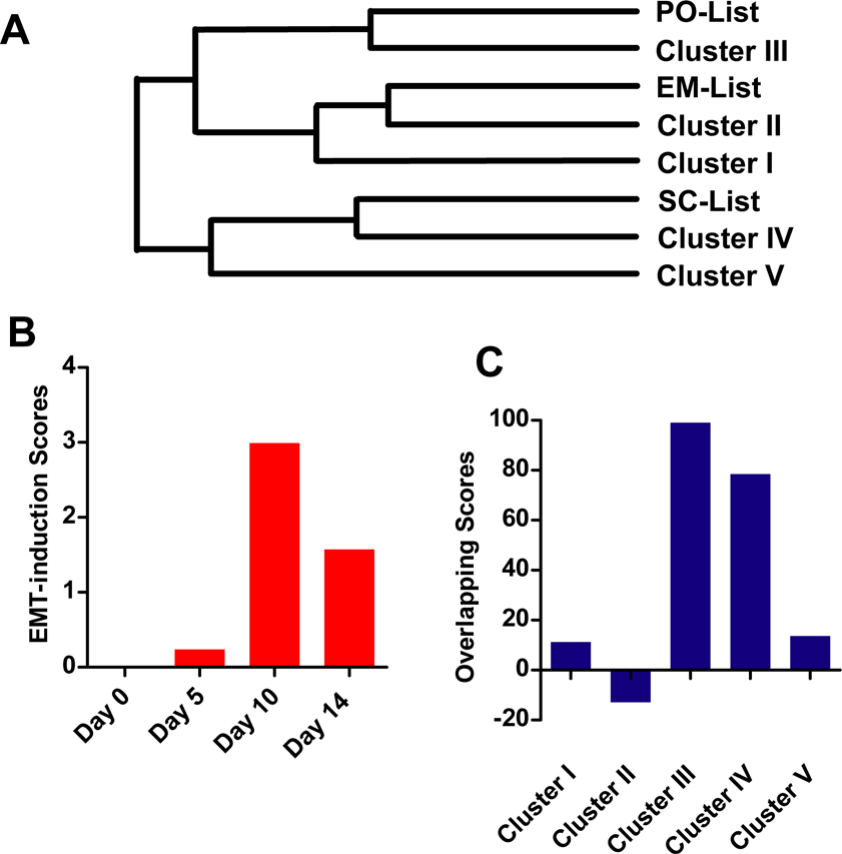
EMT during neuron trans-differentiation. (A) The 773-gene list in S5 Table was used to determine the correlation between PO-lists, EM-list, SC-list and gene lists identified in five clusters. Identification of the 773 genes in all the eight lists were remarked as “1” in S5 Table. The samples were merged in close proximity using the farthest neighbor clustering method in linkage criteria, i.e. complete linkage. (B) Four scoring systems mentioned above were used to evaluate the expression change during neuron trans-differentiation (GSE68902). (C) The expression changes on Day 10 during neuron trans-differentiation (GSE68902) were evaluated by gene listed from five clusters as described in Materials and Methods. Overlapping score was calculated by subtracting the absolute log2 values of genes with opposite expression changes from the summary of genes with consistent expression changes.

After grouping human EMT-induction samples into five clusters and identifying genes with significant expression changes, the three lists, PO-list, EM-list, and SC-list, and the five clusters were compared based on their identification with the 773 genes combined from the three lists previously (S5 Table). As indicated in Fig 6A, PO-list had a higher correlation with Cluster III, and EM-list had a higher correlation with Cluster II, while SC-list had a higher correlation with Cluster IV. Thus, although these three previous lists were intended to describe the overall changes during EMT, they were closer to certain types of EMT.

In addition, the clustering of different EMT-induction samples might help the understanding of different EMT. For example, during the trans-differentiation from MEFs to neurons, significant changes were observed on cell morphology, cell migration and genes expression [12]. By evaluating the gene expression changes during such conversion with our scoring system, an EMT was identified from Day 0 to Day 10, while a MET was identified from Day 10 to Day 14 (Fig 6B). The expression changes during the initial EMT were closer to the generic signature identified in Cluster III and Cluster IV (Fig 6C), suggesting the activation of multiple signaling pathway and specification neuron fate. Actually, EMT-induction samples in Cluster III and IV were more similar than with others (Fig 3A). Thus, EMT during neuron trans-differentiation might represent a specific type of EMT.

## Discussion

Constant efforts have been made to study the mechanism of EMT, and yet the general nature of EMT is still elusive. As revealed by this study, although the agreements between genes expression changes and pathways among different lists were low, there were still some conserved genes and pathways across different types of EMT. For example, *Cd24*, *Cdh1*, *Cdh2*, *Epcam*, *Fn1*, *Lox*, *Mmp2*, *Ocln*, *Prss8*, *Vcan*, *Vim*, and *Zeb1* were identified in PO-list, EM-list, and SC-list (Fig 1C). Most of these 12 genes were highly involved in cell adhesion and cell migration. For example, *Cdh1* and *Cdh2* are classical and the most well-studied members of the cadherin superfamily [52], and their expression ratio can be used to classify more than 80% primary cells into mesenchymal or epithelial cells [26]. *Cd24*, *Epcam*, *Fn1*, *Vim*, and *Vcan* are involved in cell adhesion and cell migration [53–55]. In addition, GO terms extracellular matrix, angiogenesis, B/T cell activation, cell adhesion and ECM-receptor signaling pathway (KEGG) were enriched in at least three of the five clusters of human samples (Fig 4). Thus, changes in cell adhesion and cell migration might be the conserved changes across multiple EMT. The low overlapping of generic signatures among the three EMT-related gene lists, on the other hand, may be resulted from the differences in the scoring systems used in the three reports. For example, in our study, the clustering of EMT samples was likely affected by types of cells lines but independent of the levels or ways of EMT induced. While according to Groger CJ, GES (gene expression studies) clustered according to the mode of EMT stimulus rather than to cell type [13]. If it is true, more reliable analysis methods may be in urgent need to better integrate and analyze EMT transcriptomes.

As a dynamic process, existence of intermediate states of EMT or uncomplete EMT has been widely accepted, reports suggested that compared to epithelial and mesenchymal states, cells at intermediate states of EMT possess the highest plasticity [56, 57] and are easier to induce or reverse EMT. Studies on the intermediate or metastable state would shed light on the mechanisms of EMT and provide guidance in clinical designs of therapeutic strategies. Tan TZ classified cells into different levels of EMT according to their proximity to epithelial and mesenchymal states, and used the level of EMT as a critical index to distinguish different EMT states [14].

The expression ratio between *Cdh1* and *Cdh2* could be used as a scoring system to evaluate EMT in this study and previous studies (S6 Table) [11, 26]. Since both genes are members of the cadherin superfamily but differ in their homotypic binding affinities and abilities to maintain cell adhesion [58, 59], the ratio between these two proteins represents the balance shift between cell adhesion and cell migration rather than a total loss of one of the two. In addition, EMT could be simply considered as the switch between Cdh1 and Cdh2 or a balance shift between tight cell adhesion and high cell mobility. The multiple rounds of EMT during embryonic development could be recognized as a route for cells to acquire high mobility and move to their desired destinations. This hypothesis is supported by the fact that cadherin superfamily is only found in metazoans except choanoflaellates with colony behavior or after the evolution from unicellularity to multicellularity [59–61]. The multicellularity not only requires cell adhesion to hold all cells together, but also requires cell migration to re-arrange certain cells to desired locations when multicellularity becomes more complex and larger. Thus the balance shift between cell adhesion and cell migration emerges soon after the evolution from unicellularity to multicellularity. In addition, as the size of multicellularity became larger and the structure became more complex, more cadherin proteins and multiple rounds of shifts were required as the multiple rounds of sequential EMT-MET during embryonic development.

If the hypothesis is true, another following hypothesis could be deduced easily. If the major function of EMT was to rearrange cells to their desired new locations by providing high mobility, it should also provide the cells the capability to convert into the desired cell types in new locations. One of the possible ways to achieve this function was to induce certain level of pluripotency or multipotency in the cells, and let them differentiate into desired cell types under certain stimulants.

EMT and MET are necessary steps in cell differentiation during embryonic development, they are also indispensable for cells to dedifferentiate into pluripotent state. Regain of pluripotency could be achieved with four transcriptional factors, Oct4, Klf4, c-Myc and Sox2 [62]. These four pluripotency-related factors regulated EMT. For example, Klf4 induces MET by direct binding to the promoter region of *Cdh1* [9]. Oct4 and Sox2 have distinct abilities to induce MET during the generation of iPSCs from MEFs [11]. In addition, all four factors except *Sox2* could be identified in SC-list [15]. Down-regulation of *Klf4* and *Sox2* were identified in Cluster II and Cluster I respectively, possibly because of their abilities to induce MET. Although not been identified in any one of the five clusters, the up-regulation of several pluripotent factors, like *Oct4*, *Nanog* and *Sall4*, were observed in about one third of the 74 human EMT-induction samples, suggesting the induction of pluripotency could be observed under certain circumstance.

However, stemness concomitant with EMT could also be considered as the emergence or increase of cancer stem cells (CSCs), which have enhanced tumorigenicity, partial stemness as in self-renewal and differentiation, and CD44^+^/CD24^-/low^ on cell surface [63, 64]. Inducing EMT in human mammary epithelial cells increased the CD44^+^/CD24^-/low^ population [31]. Up-regulation of *Cd44* was identified in 32 EMT-induction samples, while down-regulation of *Cd24* was in 45 samples, suggesting a potential increase of CD44^+^/CD24^-/low^ population (S2 Table). In addition, down-regulation of *Cd24* was the one of the twelve genes identified in PO-list, EM-list and SC-list (Fig 1C). The shared characteristics between cells undergo EMT and CSCs in TGFβ activation, circulation in blood, and chemo-resistance further suggested the possible induction of stemness by EMT or at least by EMT during cancer progression [65].

Introducing certain transcriptional factors could induce direct trans-differentiations from somatic cells to functional neurons [66, 67] and neuron stem cells. Recently, different combinations of small-molecule compounds have been reported to induce direct conversion of somatic cells into neurons [12, 68–71]. The MEFs cultured in 5C medium underwent significant morphological changes including shrinkage of cell size, increase in the number of neurite-like structures and promoted cell mobility, which suggested a conversion of MEFs to further mesenchymal state or EMT [12]. Analyzing gene expression changes during trans-differentiation with current four scoring systems further demonstrated an EMT from Day 0 to Day 10 (Fig 6B). Thus a correlation between neuron fate induction and EMT was observed. In addition, genes identified in Cluster IV of EMT-induction samples were enriched in response to vitamin A and neuron fate specification (Fig 5B), suggesting the potential ability of EMT to induce neuron fate. Such potential ability of EMT was further supported by the fact that EMT observed during neuron trans-differentiation was more similar to EMT in Cluster III and IV (Fig 6C).

Epigenetic regulations including DNA methylation, histone methylation, acetylation and so on during EMT have also been studied and reviewed extensively [16]. However, all three meta-analyses identify few genes related to epigenetic modulation. For example, *Ezh2*, *Kdm3a*, *Ctbp1* and *Bmi1* were only identified in SC-list, which was manually curated from literature and could not be considered as the generic signature for EMT. The low identification of genes related to epigenetic modulation might due to the changes in protein activity can not be revealed in microarray analysis or the expression changes of these genes are not conserved among different EMT. By grouping the 74 human EMT-induction samples into five clusters, several genes related to epigenetic modulation was identified (S9 Table). For example, significant changes of *Eed* and *Kdm2a* were observed in Cluster I. *Setdb2* and *Tet2*, *Kdm6b* and *Tet1*, *Ash1l*, *Smyd2*, and *Setd2*, *Ehmt1* and *Smyd3* were identified in listed generated from Cluster II, III, IV, and V respectively. The identification of Tet1/2 during EMT was consistent with several previous reports. In addition, Tet-mediated DNA demethylation is also essential for MET during reprogramming [20]. The correlation between Tet proteins and cancer metastasis has also been reported [72, 73].

EMT has been studied for more than decades and can be observed in a variety of biological processes. However, it is still not easy to describe the mechanisms. Even the seemingly similar stem-cell programs operating in TICs (Tumor-initiating cells) and normal stem cells of the corresponding tissue differed significantly in details[74]. Taking advantages of high throughput methods like microarray, scientists prefer to use gene expression profile to characterize different cells or different states of cells. However, it is still difficult to identify genes with conserved expression changes across EMT under different circumstances as discussed above. Thus, we hypothesized and demonstrated that there may be different types of EMT focusing on distinct pathways, and our analyses and results provided more information for related research and will facilitate the understanding of EMT.

## Conclusions

This study clustered the EMT samples into five clusters that represent five types of EMT. These five types of EMT had no difference in the intensities of induced EMT but explained the low productivity of previously identified generic signatures.

## Supporting Information

S1 Table Lists of micro-array datasets used in current analysis. 24 human and 17 mouse microarray datasets were selected and used for current analysis. The detailed information of species, GEO accession number (GEO Acc. No.), cell types, EMT inducers, platform used for micro-array analysis, number of samples under corresponding GEO accession number (Sample No.), Remarks (number of samples used, what kind of treatment was selected for analysis, and corresponding GSM number), and related publication for each dataset was included.
(XLSX)

S2 Table 74 EMT-induction samples in human. 24 human microarray datasets were merged under the official gene symbols provided by HGNC. Experiment with treatment and its corresponding control were considered as one EMT-induction pair, and the log2 values of the gene expression ratio in this EMT-induction pair were applied in the analysis. 74 human EMT-induction pairs were listed in the table.
(XLSX)

S3 Table 31 EMT-induction samples in mouse. 17 mouse microarray datasets were merged under the official gene symbols provided by MGI. Experiment with treatment and its corresponding control were considered as one EMT-induction pair, and the log2 values of the gene expression ratio in this EMT-induction pair were applied in the analysis. 31 mouse EMT-induction pairs were listed in the table.
(XLSX)

S4 Table 579 genes identified in current analysis. 579 genes which had significant expression changes in human samples and their homologues had significant changes in mouse samples were listed with official symbol, ensemble gene ID, p-values and averages. The official symbol, ensemble gene ID, p-values and averages of their homologues in mouse samples were also listed.
(XLSX)

S5 Table A 773-gene list combined from three previous lists. Genes in PO-list, EM-list, and SC-list were combined together to generate an 805-gene list. This list was further shortened to a 773-gene list because of missing in current analysis. The genes used in the four scoring systems to evaluate EMT were marked.
(XLSX)

S6 Table Different degrees of EMT induced in samples. 74 human and 31 mouse EMT-induction samples were evaluated with four scoring systems to determine the degree of induced EMT. The scores from four scoring systems and the normalized scores with average scores of human and mouse samples were provided. The classification and grouping of all the 105 samples were also listed.
(XLSX)

S7 Table Three gene lists generated after EMT evaluation. 105 EMT-induction samples were classified into three groups with distinct EMT induced, strong, medium and weak. Three gene lists were generated from these three groups of samples.
(XLSX)

S8 Table Clustering with consensus clustering method. Gene expression changes in 74 human and 31 mouse EMT-induction samples were clustered with consensus clustering method again to confirm the five clusters generated with hierarchical clustering. K was set from 2 to 10 for human or mouse samples. The resulted cluster number of provided.
(XLSX)

S9 Table Five gene lists generated from different clusters. 74 human EMT-induction samples were grouped into five clusters. Five gene lists were generated from these three five clusters.
(XLSX)

S10 Table GO enriched terms in five gene lists. 74 human EMT-induction samples were grouped into five clusters. Five gene lists were generated from these three five clusters and subjected for GO analysis. The Go terms with significant enrichment in any one of the five lists were list in the table. Some GO terms were clustered together and listed in Figs 4 & 5 with a representative GO terms.
(XLSX)
